# Physiology and Molecular Mechanisms of the “Third Fluid Space”

**DOI:** 10.3390/jcm14238491

**Published:** 2025-11-30

**Authors:** Randal O. Dull, Robert G. Hahn

**Affiliations:** 1 Department of Anesthesiology, University of Arizona College of Medicine, Tucson, AZ 85724, USA; randaldull@anesth.arizona.edu; 2 Department of Pathology, University of Arizona College of Medicine, Tucson, AZ 85724, USA; 3 Department of Surgery, University of Arizona College of Medicine, Tucson, AZ 85724, USA; 4 Karolinska Institute at Danderyds Hospital (KIDS), 171 77 Stockholm, Sweden

**Keywords:** fluid therapy, interstitial pressure, inflammation, pharmacokinetics

## Abstract

Basic physiology and molecular mechanisms accounting for the maldistribution of fluid that is characteristic of the “third fluid space” (*V*_t2_) have been known for several decades but have been poorly integrated into the clinical literature. Today, the maldistribution can be quantified and simulated in living humans by using volume kinetic mathematics, which introduces possibilities to validate interventions designed to mitigate the pathophysiology. Fluid accumulation in *V*_t2_ occurs both in fluid overload and inflammation, and both are largely influenced by interstitial fluid pressure. This is normally slightly sub-atmospheric but increases during volume loading to eventually exceed the ambient air pressure, whereby the loss of vacuum allows pools of fluid to appear in the interstitial gel. Opening of *V*_t2_ due to fluid overload can be delayed/minimized by lowering the infusion rate, hemorrhage, and the use of hyper-oncotic fluid. Accumulation of fluid in *V*_t2_ during acute inflammation and tissue injury can be explained by disruption of the cell–matrix interactions that actively regulate the interstitial pressure. Inflammatory mediators, mostly tissue cytokines, cause release of tensile forces that disrupt integrin-dependent adhesion between interstitial fibroblasts and collagen fibers. This disruption causes the interstitial space to expand, which results in a deep negative (suction) pressure. These events can be modulated by α-trinositol and insulin.

## 1. Crystalloid Fluid in Intensive Care

Intravenous administration of electrolyte-based crystalloid fluid, such as Ringer’s solution and isotonic saline, is a cornerstone in the treatment of the intensive care patient. The goal of the therapy is, together with vasoactive drugs, to maintain adequate tissue perfusion and ensure sufficient oxygenation of cells. The challenge is to achieve these goals by administering fluid in a well-timed fashion but without causing fluid overload, which promotes complications [1,2,3,4]. Intensivists are advised to increase the intravascular volume of the patients early during the onset of severe disease (“resuscitation”) and to withdraw this fluid when improvement is established (“de-resuscitation”) [5,6]. The reasons for the temporary need for volume expansion are multifactorial but altered adrenergic tone lowers the mean systemic filling pressure and redistribution of blood flows play a role.

## 2. Distribution of Infused Fluid

The crystalloid fluid circulates between the plasma and the interstitial fluid space. The fluid initially expands the plasma volume from which distribution to the interstitial space occurs by normal capillary efflux. Fluid loading increases the capillary leakage of fluid by several multiples. Its intravascular half-life is approximately 8 min, which means that the entire distribution process requires 30 min to be completed [7,8]. Animal experiments show that the lymphatic flow increases within minutes after the intravascular fluid begins to leak into the interstitium [9]. The rapid response ensures that a balanced distribution of fluid between all expandable parts of the extracellular fluid develops as soon as possible.

**Figure 1:**
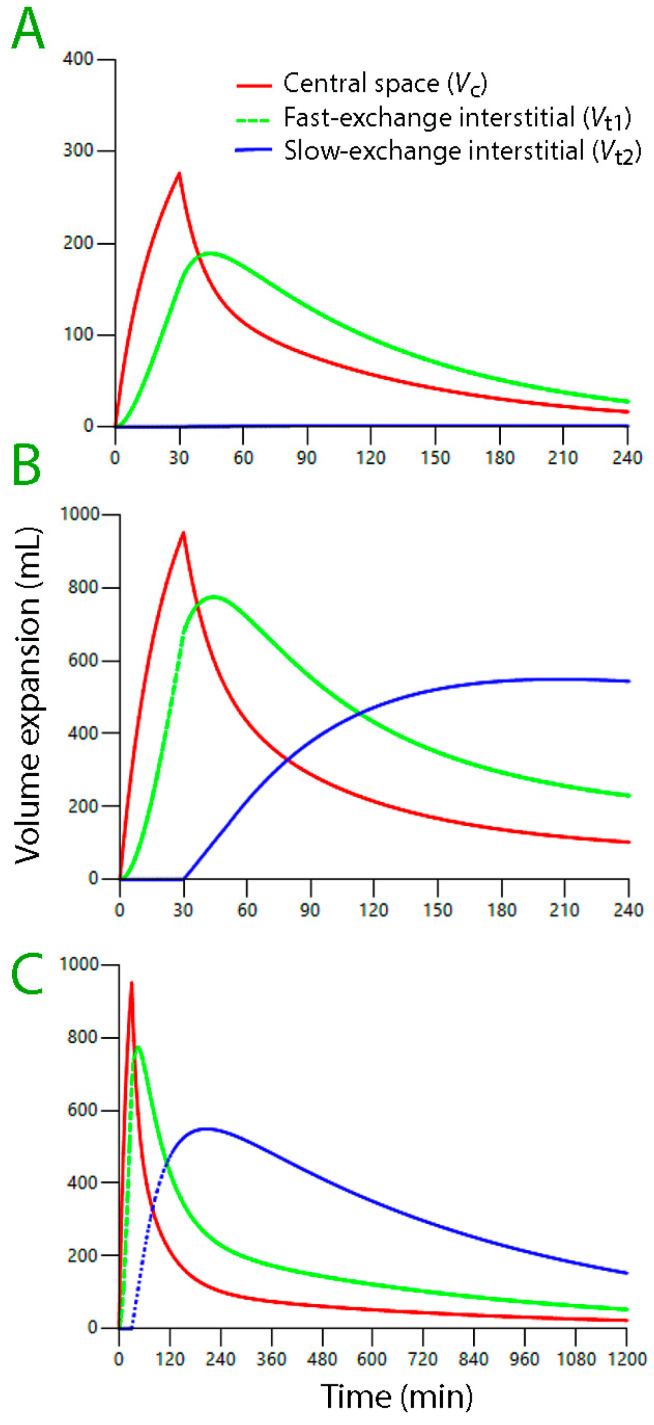
**Simulation of the distribution of Ringer’s solution in the conscious state.** (**A**) Kinetic data for 61 volunteers (mean age 44 years) who received 300–1000 mL intravenously over 30 min. Simulation is based on the mean infused volume, which was 572 mL. (**B**) Kinetic data for 105 volunteers (mean age 30 years) who received 1000–2700 intravenously over 30 min. Simulation is based on the mean infused volume, which was 1875 mL. (**C**) The time axis for simulation (**B**) is extended to 20 h. Data taken from Reference [13]. Note variable scale on the *y*-axis.

Volume kinetic analysis of the hemodilution and urine output over time can be used to illustrate how fast the equilibrating process occurs [7]. It shows that leakage of excess intravascular fluid into the interstitial space occurs approximately five times faster in a healthy awake adult compared to the accelerated urine flow, which is the primary route of elimination. Full volume equilibration of the infused fluid between plasma and the interstitial fluid space is attained 30–40 min after the end of a 30 min infusion (Figure 1A). However, when “de-resuscitation” is carried out in the severely ill, the administered fluid is difficult to withdraw actively without eliciting symptoms of hypovolemia. Hence, the “de-resuscitation” phase might need to be extended over several days, which is difficult to understand, as equilibration is normally achieved quite soon after an infusion of fluid. The answer we will advocate in this article is that both **excess fluid** or **inflammation** opens a “third fluid space” which infused fluid volumes do not normally enter. We will explore the basic science behind the “third space” which was, surprisingly, well established by physiologists several decades ago.

## 3. History of the “Third Fluid Space”

The concept of a “third fluid space” was introduced in the 1960s as a secondary interstitial space that becomes filled with fluid during trauma and major surgery [10,11]. However, the belief that this space should be filled when performing fluid therapy encouraged dramatic increases in the volumes used, which promoted respiratory distress syndromes and other complications. A more moderate approach was soon called upon. Lack of hard evidence further excluded “the third space” from medical research and teachings in anesthesia and intensive care during the subsequent 50 years. However, volume kinetic analysis has recently been able to demonstrate, by studying large number of humans using mixed-model mathematics, the existence of a “third fluid space” that is involved in the distribution of crystalloid fluid under special circumstances [12,13].

In the complete volume kinetic model, crystalloid fluid is infused into a central volume *V*_c_ (the plasma) and then distributes to a fast-exchange interstitial space, *V*_t1_, and further to the “third fluid space” which is from now on denoted as “the slow-exchange interstitial space”, *V*_t2_. It is currently believed that *V*_t1_ corresponds to the free-water channels of the interstitium [14,15] and the lymphatic vessels [16], while the slow-exchange compartment likely corresponds to the interstitial gel phase, where the flow of fluid is known to be restricted [17,18].

Basic research efforts in physiology have outlined the circumstances under which fluid accumulates in *V*_t2_ in settings of volume overload and acute systemic inflammation. The first mechanism was explored by the American physiologist Arthur Guyton in the 1960s and the second was studied in the 1990s by the Norwegian group of Wiig, Reed, and Lund. The following paragraphs summarize their key findings.

## 4. Guyton’s Studies of P_if_

Arthur Guyton and co-workers performed a series of studies in dogs where he measured the interstitial hydrostatic pressure, P_if_, using different methods. Surprisingly, P_if_ appeared to be slightly sub-atmospheric by −2 mmHg to −6 mmHg [19,20]. This negative pressure holds the tissues together and limits fluid movements. Guyton found that P_if_ slowly increases when fluid is infused. When P_if_ reaches zero, he recorded a massive increase in the interstitial compliance, whereby large amounts of fluid could enter the interstitium without being markedly restricted by a counterpressure from the tissues.

These findings correspond well in that *V*_t2_ in living humans opens for volume expansion when *V*_t1_ has increased by 600–800 mL, which normally occurs when 1.2–1.3 L of Ringer over 30 min has been infused in a healthy human [13] (Figure 1B). Opening of *V*_t1_ occurs suddenly and probably operates as an overflow reservoir. The turnover of the fluid in *V*_t2_ is very slow which markedly prolongs the half-life to the fluid in the body (Figure 1C). The volume does not increase indefinitely but stops when the expansion amounts to 1/3 of the infused volume. However, the gel tissue loses elasticity over time and a study in septic pigs suggest that the volume of *V*_t2_ the next day might amount to 10% of the body weight; further interstitial expansion seems to occur in *V*_t1_ [21].

***Capsule and needle techniques.*** The ability to measure P_if_ is central to our understanding of why *V*_t2_ becomes filled with fluid under certain conditions. Guyton and colleagues, over the span of several decades from the 1950s through the 1970s, contributed greatly to our understanding of P_if_ [22]. Early attempts to measure P_if_ used needles (usually 22–25 gauge) connected to a pressure transducer but failed due to the size of the needle tip relative to the small, free-water channels in the interstitium. Needle insertion caused bleeding and tissue trauma which occluded the needles, and the large tip impacted solid components of the interstitial space thus, measuring solid pressure mechanics rather than free-fluid pressure [23].

To overcome the obstacles for measuring P_if_ with needle techniques, Guyton and colleagues developed the perforated capsule method which allowed for repeated measures over a time range of weeks [19]. Briefly, small, perforated capsules (sizes tested ranged from 0.5 cm up to 2 cm) composed of a variety of plastics or metal were implanted into the subcutaneous tissue of experimental animals. A small needle was inserted into the skin and through one of the perforations into the capsule to measure P_if_. It was hypothesized that the interior fluid pressure of the capsule would be in dynamic equilibrium with P_if_ outside the capsule, and the rigidity of the capsule prevented atmospheric pressure from influencing the measurements.

Guyton described the incorporation of the capsule into the interstitial space and the effect on P_if_. Immediately after surgical insertions, the capsule contained air and some fluid that precluded immediate use. Over the course of hours to days, tissue inflammation occurred that filled the capsule with fluid, while the surrounding edema produced a positive P_if_ from within the capsule. After several weeks, as the inflammation subsided, the internal pressure became increasingly more negative and stabilized at what was assumed to be normal tissue P_if_. Using this technique, Guyton found that P_if_ was stable at approximately −5 to −6 mmHg [19,24].

***P_if_ and the Starling forces.*** To validate the dynamics and sensitivity of the internal capsule pressure in terms of local Starling forces, Guyton tested multiple interventions which included: venous occlusion; arterial occlusion; rapid changes in plasma oncotic pressure, and rapid plasma volume expansion [19]. The results of these studies are as follows. An acute increase in venous pressure (+50 mmHg) caused an immediate and sustained increase in capsular P_if_ from −7 mmHg to 0 mmHg over a period of 2 h. The immediate increase in capsular P_if_ indicated that fluid inside the capsule was in dynamic equilibrium with tissue pressure. Similarly, acute arterial occlusion resulted in decreased capillary pressure and shifted Starling forces towards reabsorption; capsular P_if_ became more negative by an average of −1.4 mmHg, consistent with fluid reabsorption. Rapid administration of hyper-oncotic dextran 20% into the vascular compartment (increased plasma osmotic pressure, favoring fluid reabsorption) resulted in an immediate and deep decrease in P_if_ to more negative values, reaching −15 mm Hg within minutes. Lastly, large volume infusion of crystalloid fluid over several hours produced significant tissue edema, resulting in an increase in capsular pressure from −3.2 mmHg to +7 mmHg. In all interventions, capsular P_if_ changed almost immediately and in accordance with changes in local Starling forces and providing strong evidence that the P_if_ of the perforated capsules reflected local tissue P_if_.

## 5. Wiig’s Microneedles

In the 1980s, Wiig and colleagues developed a technique using glass microneedles (pulled glass micropipettes) connected to a servo-controlled pressure transducer to measure P_if_ in experimental animals [25]. The microneedle tip diameters were 2–4 μm and could be inserted in the skin to a depth of 0.1–0.5 mm; Wiig and colleagues consistently measured P_if_ in healthy tissues of −2.0 ± 0.6 mmHg [26], which is higher than Guyton’s perforated capsule measurements. An explanation for the differences for the absolute value of baseline P_if_ using different methods include the depths where P_if_ was measured: capsules were implanted deeper in the subcutis layer, whereas microneedle measurements were made in the more superficial reticular and papillary layers of skin [27]. Wiig and colleagues also postulated that that state of hydration of the experimental animals also influence P_if_ measurements.

Despite the method-dependent differences in absolute P_if_, a consistent biomechanical picture of the interstitial space emerges. Pressures are negative (sub-atmospheric) at baseline, and pressure–volume curves demonstrate that the compliance of the interstitial space is very low and small changes in free-water volume result in a steep positive rise in P_if_. Similarly, the pressure–mobility relationship demonstrated that the interstitial space offers a very high resistance to free-water movement when P_if_ is sub-atmospheric but, when P_if_ reaches zero and above (positive values), resistance to water movement decreased by more than a 100,000-fold [20]. Using the pressure–volume relationship in concert with conductance data suggests that the amount of free water is very small, approximately 1%. This means that most of the water in the interstitial space is bound within the gel phase, composed primarily of hyaluronan and other glycosaminoglycans.

## 6. Biomechanics of the Interstitial Space

***The interstitium is dehydrated.*** Under control conditions, the interstitial space exists, biomechanically, in a contracted state, created by the tethering of interstitial cells with the extracellular matrix including collagen, laminin and other filamentous proteins that provide structure to the space. The adhesion tension between the cells and matrix fibers keeps the interstitial space held at a minimum volume (small free-water volume) and the tensile forces maintain P_if_ at values ranging from −2 to −6 mmHg [19,26]. The identity of the interstitial cells that participate in the cell–matrix binding have not been clearly delineated, but they are usually referred to as interstitial fibroblasts. They lack specific molecular markers to be easily categorized [14] but, for the purposes of our discussion, we will refer to them as fibroblasts. They are positioned on only one side of a collagen fiber, suggesting an arrangement to produce an organized and directional tension [14].

***Role of integrins.*** Interstitial fibroblasts bind to collagen and other matrix fibers via cell-surface adhesion molecules called *integrins*. Collagen-binding integrins include α_1_β_1_, α_2_β_1_, α_10_β_1_ and α_11_β_1_ and it appears that basal tension is mediated primarily by the β_1_-integrins [28]. The details of integrin-dependent adhesion to matrix proteins have been worked out using the collagen-contraction assay where fibroblasts are seeded into collagen gel. Over time, the cells impart tension onto the collagen gel resulting in a contraction and compaction of the gel. Anti-integrin antibodies and integrin knock-out cells have aided in characterizing the cell–matrix interactions and successful gel-relaxation strategies found in vitro can then be validated in vivo using techniques to measure P_if_ [28].

Disruption of the integrin-dependent adhesion between interstitial fibroblasts with collagen and other matrix proteins results in untethering and a rapid expansion of the interstitial space; this rapid expansion creates an acute increase in negative pressure, e.g., “suction pressure” as low as −150 mmHg [29,30]. The rapid, highly negative suction pressure will pull large quantities of fluid across the capillary wall and into the interstitial space.

As previously reviewed, under basal conditions, the interstitial space is held in a compacted state and the amount of free water is minimal and water mobility is low [20]. The bulk of interstitial water appears to be held within the abundant hyaluronan gel-phase, which represents bound water that has little if any mobility. Any expansion of the interstitial space increases the mobility of free water but can also be predicted to increase access of free water to the gel phase. This seems to be more important when capillary hydrostatic pressure increases, as during volume overload, and the increased interstitial fluid volume, and, specifically, increased gel-associated water content, may function as a reservoir to aid in buffering circulatory overload [22].

## 7. Two Patterns for P_if_ and Volume Changes

There are two mechanisms that can alter the interstitial fluid volume and, hence P_if_: we can call them “push vs. pull”.

### 7.1. Hydrostatic Mechanism: Elevated Capillary Hydrostatic Pressure Pushes Fluid from the Vascular Compartment into the Interstitial Space (Monophasic Pressure Change)

Acute increases in capillary and/or venous hydrostatic pressure will increase fluid filtration across the endothelial barrier, even without changes in endothelial junctional permeability. The interstitial space has a low compliance at its basal state and any increase in free fluid will significantly increase P_if_ towards more positive values. As the interstitial volume increases, fragmentation of matrix elements occurs, contributing to a disruption of the interstitial architecture, allowing for increased fluid mobility [31,32]. When the volume of fluid entering the interstitial space drives P_if_ towards 0 mm Hg and above, there is little resistance to fluid entrance as compliance has risen significantly and, thus, fluid mobility rises dramatically [20].

### 7.2. Inflammatory Mechanism: Acute Negative Interstitial Pressure Pulls Fluid into Interstitial Space; Increasing Volume Causes a Rise in P_if_ (Biphasic Pressure Change)

During acute inflammation and tissue injury, cytokine-mediated inhibition of cell–matrix interactions results in an acute release of tensile forces, causing the interstitial space to expand, resulting in a deep negative pressure (suction pressure). It is likely that acute inflammatory processes are also associated with increased protease activity that contributes to matrix disruption which also contributes to the acute expansion and deeply negative P_if_. For example, during burn injuries, P_if_ has been measured to be as low as −150 mm Hg [29,30]. This suction pressure rapidly pulls large volumes of fluid from the vascular compartment into the interstitial space. The increase in interstitial volume then drives P_if_ towards positive values in relationship to the new pressure–volume relationship. Inflammatory mediators inhibit lymphatic pumping so there is a concurrent reduction in fluid outflow and this lymphatic dysfunction contributes to interstitial overload. Fluid will continue to enter the interstitial space until P_if_ rises toward 0 mm Hg. Thus, the inflammatory response is associated with a biphasic pressure response.

## 8. Pharmacological and Physiological Modulation of P_if_

Various agonists and anti-integrin antibodies have been tested in vivo, using micropipette techniques, to measure their effect on P_if_. Agonists that can prevent the negative pressure associated with acute inflammation have the potential to prevent edema and, therefore, would have numerous clinical applications. Agents that have been identified as increasing P_if_ (e.g., more positive P_if_) act by increasing cell–matrix tension and include platelet-derived growth factor BB (PDGF-BB) [33], insulin [34], PGF_2α_ [35], and α-trinositol [36].

***Trinositiol.*** The first agonist reported to exhibit edema-preventing activity was α-trinositol (1,2,6-D-myo-inositol trisphosphate), a substance that is derived from phytic acid [36]. It has been reported to have anti-inflammatory activity as an inhibitor of neuropeptide-Y [37] as well as stabilizing β_1_-integrin activity [34]. An injection of α-trinositol into the rat dermis prevented the lowering of P_if_ in response to anti-β1 integrin antibodies. The effects of α-trinositol were tested both in vivo and in vitro with similar effects; α-trinositol inhibited fibroblast/collagen-gel contraction and was additive to the effects of PDGF-BB on in vivo P_if_. Dibutyryl-cAMP, when injected into the dermis of a rat, produced a negative P_if_ and edema; α-trinositol inhibited both actions of dibuturyl-cAMP, suggesting that α-trinositol acted through intracellular signaling pathways.

***Intracellular mechanisms.*** Studies into the intracellular mechanism(s) that promote cell–matrix interactions demonstrated that phosphotidyl-3-kinase and calcium are key regulators [38]. The contraction induced by PDGF-BB on fibroblast-collagen gels was inhibited by wortmannin and LY294002, both PI-3-kinase inhibitors. When tested in vivo, wortmannin caused a more negative P_if_, which was attenuated by α-trinositol, suggesting that phosphotidyl-3-kinase activity is required to maintain normal P_if_ and establish another target for α-trinositol. When the PI-3Kinase binding site for the PDGF receptor was mutated in stem cells, they lost their ability to contract collagen gels in response to PDGF-BB. When studied in vivo, mast cell degranulation led to an acute lowering of P_if_ to more negative values. This change was reversed by PDFG-BB in control mice; however, this reversal was lost in the PI-3-kinase mutant mice [39]. These results demonstrate the importance of PI-3-kinase in actively modulating P_if_.

***Ischemia–reperfusion.*** Using an ischemia–reperfusion (IR) model based on rat hindlimb occlusive tourniquet for 2 h, Nedrebo et al. [40] reported that IR injury induced a 10-fold decrease in P_if_ from a baseline of 0.5 mm Hg to −5.0 mmHg and caused a 20-fold increase in albumin extravasation. Alpha-trinositol abolished the reduction in P_if_ and reduced albumin extravasation to a 2-fold increase from baseline. These results support the previous studies demonstrating that α-trinositol stabilizes dermal fibroblast β_1_-integrin activity to maintain P_if_ and prevent negative pressure edema.

***Insulin and integrins.*** Insulin has been reported to have anti-inflammatory effects [41] and, notably, following insulin binding to its receptor, it complexes with phosphotidyl-3-kinase [42]. Importantly, insulin-dependent activation of PI-3K stimulates integrin-dependent cell adhesion [43], suggesting that insulin may also participate in regulation of P_if_. This hypothesis was tested by Nedrebo et al. [40] who reported that intravenous administration of LPS, TNFα and IL-1β all caused an acute lowering of P_if_ in rat dermis. An injection of insulin into the dermis reversed the increased negative pressure towards normal. When insulin + wortmannin were injected together, the effects of insulin were attenuated, supporting the role of insulin in regulating P_if_ via PI-3 kinase.

Further studies on insulin and P_if_ were undertaken by Svendsen et al. in 2008 [44]. Using C57black mice, they reported that lipopolysaccharide (LPS) lowered P_if_, from −0.2 mm Hg to −1.6 mmHg, an 8-fold change. When insulin was injected after LPS, the change in P_if_ was reduced by half, from −0.2 to −0.8 mmHg. In the same study, when b_3_-integrin-deficient mice were used, LPS reduced P_if_ from −0.2 to −1.5, but the reversal of P_if_ by insulin was lost. This was the first study to suggest that basal P_if_ was set by β_1_-integrin-mediated cell–matrix interactions. The reversal of inflammatory-induced reduction in P_if_ seems to be mediated by α_v_β_1_-3-integrins [28].

Further support for the role of β_3_-integrins in the reversal of P_if_ during inflammation was provided by Linden et al. [33]. Using a model of anaphylaxis, PDGF-BB was able to reverse the effects of mast cell degranulation on P_if_. Anti-β_3_ integrin antibodies and an arginine-glycine-aspartic (RGD) peptide to β_3_-integrin inhibited the effects of PDGF-BB on reversing P_if_ following anaphylaxis. Similar results were obtained using the fibroblast-collagen contraction assay, with _v_-β_3_-integrin-deficient fibroblasts and PDGF-BB. Collectively, these results suggest that β_3_-integrins are major participants in the reversal of inflammatory-induced reduction in P_if_.

***Osmotic pressure.*** Arturson and Mellander [45] reported that during second-degree scald injury, the capillary filtration coefficient increased 2–3-fold, but the edema volume would have required a change in permeability in excess of 15-fold. To understand the driving force for such extensive edema, they measured the osmotic pressure of interstitial fluid collected from the burn area, and it was found to be 374–476 mmHg. It can be assumed that burn-induced fragmentation of interstitial matrix elements and release of intracellular contents contributed to the change in tissue osmotic pressure. This is one example of how factors other than endothelial permeability, per se, contribute to edema formation and are, in fact, quantitatively more important. Deep negative changes in P_if_ increase interstitial fluid osmotic pressure.Inflammatory-induced inhibition of lymphatic pumping [46] further contributes to edema formation.

**Figure 2:**
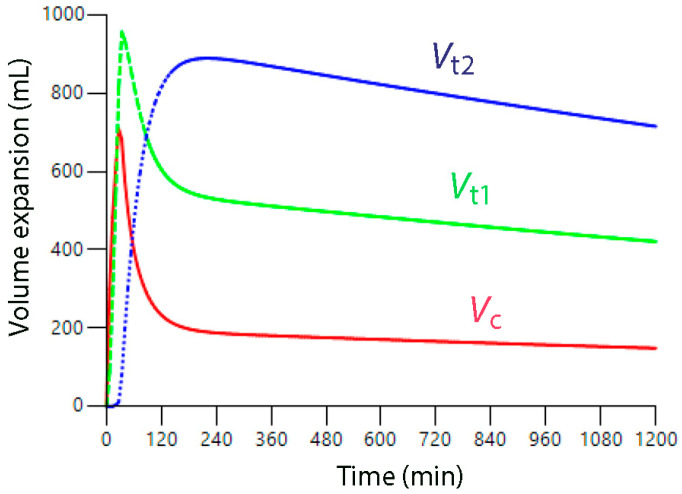
**Simulation of the distribution of Ringer’s solution during general anesthesia.** Fluid infusion of 1875 mL over 30 min (cf. Figure 1C). The basic differences in fluid kinetics between the conscious and the anesthetized states consist of a marked reduction in the rate constant for urine flow (−80 to −90%) [7] and inhibition of the rate constant for return flow of filtered fluid to the plasma (−30 to −50%), i.e., the lymphatic flow, which is due to both the mechanical ventilation and inhibitory influences of anesthetics on lymphatic pumping [16]. Kinetic data were taken from Reference [13]. Measurements lasted for 150 min but the curve is extended to 20 h to predict the course over time.

**Figure 3:**
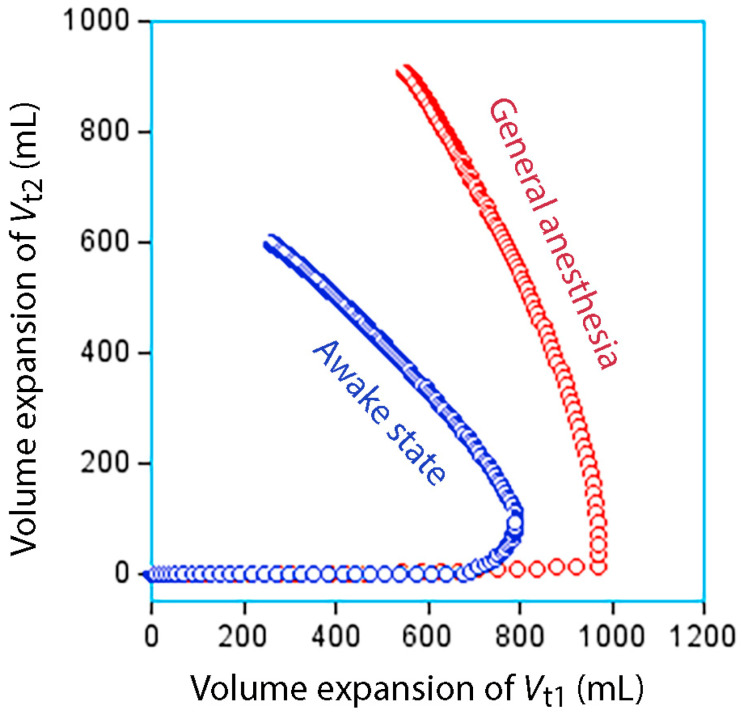
**Relationship between *V*_t1_ and *V*_t2_ in awake and anesthetized states.** Simulated volume expansion in the fast-exchange (*V*_t1_) versus slow-exchange (*V*_t2_) fluid compartments every minute during 3 h when 1875 mL of Ringer’s is infused over 30 min in the awake and anesthetized states. Opening of *V*_t2_ opens later during anesthesia due to redistribution of 200–300 mL of interstitial fluid to the plasma when anesthesia is induced. The longer duration of the expansion of *V*_t2_ during anesthesia is due to the inhibition of the urine flow and the retardation of the lymphatic flow. Kinetic data was taken from Reference [13].

***General anesthesia.*** The anesthetized state is characterized by a marked inhibition of the diuretic response to fluid (−80% to −90%) [7], faster distribution of infused fluid from the plasma to the interstitium [8] and retarded lymphatic flow [16]. These kinetic differences from the awake state make elimination of infused fluid more difficult and could even create a long-standing overloading situation (Figure 2). However, induction of general anesthesia is associated with a redistribution of 200–300 mL interstitial water due to the decrease in the arterial pressure [47] which decreases P_if_. Therefore, slightly more infusion fluid can enter *V*_t1_ before *V*_t2_ opens during general anesthesia, although the expansion is more persistent due to retarded lymphatic and urine flows (Figure 3).

***Miscellaneous.*** All events and manipulations that selectively redistribute interstitial fluid to the plasma by capillary refill will delay, or even prevent, opening of *V*_t2_ due to the “push” mechanism. These factors include hemorrhage and the use of hyper-oncotic infusion fluid [48].

Stimulation of α_1_-adrenergic receptors increase lymphatic return of interstitial fluid [49,50,51] which aids in keeping *V*_t1_ small. By contrast, β_1_-adrenergic receptors inhibit lymphatic pumping and thus reduce the fluid clearance from the interstitial space [16,51]. During sepsis, vasopressors have relatively little effect in the maldistribution of fluid into *V*_t2_, suggesting that adrenergic agonists cannot restore adequate lymphatic pumping in the setting of acute inflammation [51].

Opening and filling of *V*_t2_ due to the “push” mechanism can also be delayed or prevented by infusing fluid at a lower rate. The reason is that more of the infused volume then has time to become excreted [13]. The prevention works well in awake subjects while the low renal clearance for fluid during anesthesia makes this strategy largely inefficient.

The opposite manipulations are likely to limit the filling of *V*_t2_ if the third-spacing of fluid is due to the “pull” mechanism because P_if_ is then low instead of high.

## 9. What the Physiology Tells the Clinician

This previous section demonstrated that the interstitial space is actively regulated through intercellular pathways that determine fibroblast-matrix adhesion and, subsequently, cell-mediated matrix tension. The ability of α-trinositol and insulin to reverse inflammatory-induced lowering of P_if_ and the associated negative-pressure interstitial edema should not be lost on clinicians, specifically anesthesiologists and intensivists, who often strive to maintain hemodynamic stability without fluid-overloading patients, which can result in peripheral edema. The validation of efficacy and safety for drugs targeting the active regulation of P_if_ are needed in many aspects of clinical medicine.

The recognition that P_if_ is actively regulated by cell–matrix interactions and can increase to large negative pressures clarifies historical difficulties in accounting for the magnitude of edema that develops when the change in capillary permeability is considered as the principal mechanism. The net filtration pressure in normal dermal and skeletal muscle tissue is low, only 0.5 to 1.0 mmHg [52]. At this low filtration pressure, the modest changes in capillary filtration coefficient would be insufficient to account for the observed change in the volume of interstitial fluid. For tissue edema to develop visibly, it has been estimated that capillary fluid filtration would have to increase more than 100-fold [38] and, therefore, requires and different mechanistic explanation.

## 10. What Volume Kinetics Adds

The works by Guyon’s group and Wiig, Reed & Lund explored the conditions under which excessive accumulation of fluid occurs in the interstitial space, but they did not consider it to represent a separate fluid space. Recent compartmental analyses by volume kinetics demonstrate that a “third fluid space” indeed has characteristics that differ from the fast-exchange fluid space (*V*_t1_) that is situated closer to the circulating blood.

The size of *V*_t1_ is approximately twice the plasma volume and consists of, as it appears in our kinetic analyses, free-flowing fluid. Recent morphological studies show that interstitium contains a widespread web of water channels that can transport water over long distances [14,15], and those were not known when Guyton performed his studies. These channels are likely to be part of *V*_t1_ together with the lymphatic vessels. The return of filtered fluid to the plasma increases in direct proportion to the filling of *V*_t1_, which agrees well with the known relationship between interstitial volume and lymphatic flow [52].

Data on the body fluid volumes and flow rates involved in these circuits can be obtained from a recent study where between 500 and 1000 mL of Ringer’s was infused in a subgroup of 23 volunteers [13]. The central space (*V*_c_) was 3.4 L and the fast-exchange space 7.4 L. At baseline, the capillary leakage rate of 7 mL/min [8] makes a transit time of 17.6 h. With a plasma volume expansion of 300 mL, the capillary leakage rate increases by 11 mL/min and the transit time is then shortened to 7.3 h [7400/(7 + 11) min].

By contrast, the size of *V*_t2_ is often supra-physiological, i.e., larger than the known interstitial fluid space, which suggests that the flow is not free but bound within the gel phase. In the same study as the one cited above, a kinetic analysis was made of 38 infusions where between 2.0 and 2.7 L of Ringer’s solution had been administered to volunteers over 30 min [13]. The size of *V*_t1_ was 7.6 L and the transit time 5.7 h at a constant plasma volume expansion of 300 mL. However, some of the fluid entered the *V*_t2_ compartment, which had a volume of 28 L (sometimes the size can reach 100 L). Here, the transit time is 51 h. Hence, opening of *V*_t2_ will considerably prolong the period of time when infused fluid remains in the body. The clinical observation that “de-resuscitation” takes a long time (days) to be completed can be understood from the long transit time of fluid in *V*_t2_.

The late predominance of *V*_t2_ over the other two fluid compartments (Figure 1C) and its long transit time are probably due to the fact that the flow from *V*_t2_ to *V*_t1_ is not primarily governed by hydrostatic forces. We hypothesize that the fragmentation of the interstitial matrix and the integrin-mediated cell–matrix interactions must be restored before *V*_t2_ can be “closed” and operate as intended. A normal steady-state capillary filtration and balanced lymphatic flow are also needed to create the dehydrated state of the interstitium that achieves and maintains its slightly sub-atmospheric pressure.

Guyton’s calculations suggested that the filling of the interstitium due to the “push” mechanism could rapidly amount to many liters once *V*_t2_ was opened [53]. However, volume kinetic studies indicate that only approximately 1/3 of the infused volume expands *V*_t2_ during the acute phase, although “stress relaxation” might allow further expansion if intravenous volume administration continues to be ambitious [54]. Opening of *V*_t2_ means that pools of free fluid (lacunae) appear in the tissues, as has been shown in overloaded animals [55,56].

The filling of *V*_t2_ also seems to occur more slowly than the almost instant and sharp increase in the interstitial compliance for volume expansion that is implicated by Guyton et al. [20] (Figure 1). This difference is probably due to uneven distribution of infused fluid due to variability in interstitial compliance for volume expansion. Therefore, *V*_t2_ might open at different points in time in different body regions—the kinetic method models the average.

A common clinical picture in the ICU is the combination of hypovolemia, hypoalbuminemia, and peripheral edema [46]. It is likely that accumulation of fluid in *V*_t2_ is a central problem in the creation of this triad. There is a competition between the flow of fluid via the lymphatics (via *k*_21_) and transfer to *V*_t2_ (via *k*_23_), which means that the plasma will receive less return flow of fluid and albumin in relation to the filtered amounts, creating an unbalanced situation. This is most problematic when filling of *V*_t2_ is due to the “pull” mechanism because the *k*_21_/*k*_23_ ratio is then <1 [51].

The lymphatic flow becomes very low if P_if_ falls below −6 mmHg [53], which promotes hypovolemia and hypoalbuminemia more strongly than third-spacing due to fluid overload.

## 11. Questioning the Role of the Glycocalyx

Our emphasis on the physiology of the interstitium and pathological variations in P_if_ to explain peripheral edema might seem to be a departure from the contemporary understanding of the endothelial glycocalyx layer as being the regulator of fluid distribution. A healthy balance between capillary filtration, P_if_, and lymphatic pumping is needed to prevent edema. Under most conditions, the lymphatic system is the primary driver for maintaining interstitial protein and fluid balance. The view that glycocalyx damage alone explains edema is probably an oversimplification that does not agree well with experimental and clinical data.

For example, Weinbaum and colleagues [57] have stated that the measured fluid conductance (Lp) of the capillary wall limits the thickness of the glycocalyx overlying the cell junction to approximately 150 nm. In the region of the junction, the glycocalyx is distinctly different than the 1–2 mm thick structure found over the cell body. In studies of glycocalyx shedding (hemorrhagic shock, post-burn), marked increases in plasma glycocalyx fragments were not associated with changes in fluid or protein permeability [58,59,60].

To further confound the role of glycocalyx damage on capillary permeability, Zhang et al. have demonstrated that the heparan sulfate proteoglycans in the glycocalyx signal via STAT3 to endothelial tight junctions; degradation of heparan sulfates act to open tight junctions, resulting in increased paracellular permeability [61]. Similarly, Jannaway et al. reported that fragments of syndecan−3 and −4 ectodomains induce rapid disassembly of VE-cadherin leading to increased junctional permeability to albumin [62]. It appears that glycocalyx degradation is closely associated with increased junctional permeability, thus casting doubt on the specific role of the glycocalyx as the primary permeability barrier [63].

The use of frogs as the basis for assessing capillary permeability and interstitial physiology has some drawbacks. Anurans have very high interstitial compliance, unique from other vertebrates, that allows the storage of large volumes of lymph that can be mobilized during periods of dehydration [64]. Similarly, frogs can mobilize interstitial fluid by the lymphatic system at rates 10-fold higher than mammals [64] which may explain the lack of observed fluid reabsorption [65]. Adamson et al. [66] reported similar findings regarding the difference in fluid distribution between transient and steady-state conditions, compared to frogs, on fluid reabsorption. Interestingly, rat mesenteric P_if_ did not change during prolonged levels of filtration [67]; we can only speculate that mesenteric microvessels have a distinctly different interstitial compliance relative to the major fluid reservoirs in mammals, specifically skin and skeletal muscle, which have low compliance and steep pressure–volume curves [28,68].

Our studies of settings with peripheral edema in large animals or humans do not support that the flow of fluid from the plasma to the interstitium is accelerated; the kinetic data rather show that *k*_21_, which represents the lymphatic flow rate, is very low [46,51,69,70]. The lymphatic system has a key role in maintaining the normal fluid distribution. Its effectiveness in clearing the interstitium from fluid overload is markedly impaired by inflammation-induced reductions in P_if_ but the pumping activity is also directly inhibited by these inflammatory mediators, particularly by nitric oxide. By contrast, a solitary increase in the capillary filtration is not enough to create edema as it quickly accelerates the lymphatic pumping [9]. The increased turnover rate of the interstitial fluid pool even causes a slight increase in the intravascular content of albumin, which is a phenomenon that AC Guyton called “interstitial washdown” [71].

## 12. Conclusions

Fluid overload appears to open the “third fluid space” (*V*_t2_) by increasing the interstitial pressure (P_if_) to reach and exceed the atmospheric pressure. Inflammatory conditions can also open *V*_t2_, but by lowering P_if_ enough to create a “suction pressure”. This is due to vasoactive mediators that disrupt integrin-mediated cell–matrix interactions, whereby fluid is allowed to enter the gel phase and be reversibly bound inside *V*_t2_. Volume kinetic analyses show that opening of *V*_t2_ greatly prolongs the half-life of infused fluid in the body and suggest lack of free flow of fluid in the “third space”. These two mechanisms have so far only been explored in physiology and molecular biology but can today be analyzed and studied in living humans by using whole-body kinetic modeling. The possibility of analyzing and simulating “third-spacing” allows studies to be performed that modify the pathophysiology.

## Data Availability

No new data were created or analyzed in this study. Data sharing is not applicable to this article.
